# Respiratory Uptake and Depuration Kinetics of Perfluorooctanesulfonate (PFOS) in a Marine Sandworm Species

**DOI:** 10.1007/s00128-017-2124-4

**Published:** 2017-06-20

**Authors:** Takeo Sakurai, Jun Kobayashi, Nozomi Ito, Shigeko Serizawa, Hiroaki Shiraishi, Tohru Yabe, Yuichi Ishii, Noriyuki Suzuki

**Affiliations:** 1 National Institute for Environmental Studies, 16-2 Onogawa, Tsukuba, Ibaraki 305-8506 Japan; 2 Prefectural University of Kumamoto, 3-1-100 Tsukide, Kumamoto, Kumamoto, 862-8502 Japan; 3 Tokyo Metropolitan Research Institute for Environmental Protection, 1-7-5 Shinsuna, Koutou, Tokyo, 136-0075 Japan

**Keywords:** Bioconcentration, Bioaccumulation, Persistent organic pollutants (POPs), Absorption efficiency, Perfluoroalkyl acids, Polychaete

## Abstract

We determined the respiratory uptake and depuration kinetics of perfluorooctanesulfonate (PFOS) in *Perinereis wilsoni*, a polychaete sandworm used as a model species to investigate the fate of chemical pollutants in coastal environments. The sandworms were kept in gravel-packed containers, and the water levels were varied cyclically to mimic the tides. We used seawater kept at 17.1°C. A 7-day exposure period was followed by a 9-day depuration period. The dissolved PFOS concentration averaged 28 ng/L during the exposure period. Sandworm samples were collected regularly for analysis of PFOS concentrations, and a first-order-kinetics model was applied to the concentrations. The respiratory absorption efficiency of PFOS was estimated to be 11% that of oxygen, which is higher than the corresponding estimates reported for several fish species. The estimated depuration half-life of 15 days was comparable to previously reported estimates for fish and oligochaete species. The bioconcentration factor was 470.

Bioaccumulation of perfluoroalkyl acids (PFAAs), including perfluorooctanesulfonate (PFOS), in aquatic organisms is of interest because these ionizable compounds persist in aquatic environments (Giesy and Kannan 2002) and because consumption of seafood is a major source of human exposure to PFAAs (Haug et al. 2010). The transfer of chemicals to aquatic organisms in marine environments deserves further investigation (Sakurai et al. 2013) because marine fish and shellfish globally account for more than half of the production from the fishery industry and of the caloric intake from aquatic organisms. A limited number of taxa have been covered by bioaccumulation kinetics studies (Kobayashi et al. 2013), and a basis for interspecies extrapolation of bioaccumulation kinetics has not been established. Invertebrates, including polychaetes, are important prey of fish and shellfish in coastal and shallow marine environments and thus represent an important route for the transfer of chemicals to higher trophic levels (Reynoldson 1987). However, the bioaccumulation kinetics of PFAAs in invertebrates, and polychaetes in particular, has not been extensively studied. The polychaete sandworm *Perinereis wilsoni*, also known as *P. nuntia vallata*, is commonly found in gravel in intertidal reef flats or rocky shores in East Asia, and other *Perinereis* sandworms are distributed globally (Glasby and Hsieh 2006; Imajima 1972). *P. wilsoni* has previously been used as a model species for investigating the fate of chemicals in coastal environments (Kono et al. 2010; Nurulnadia et al. 2013). In this study we determined the respiratory uptake and depuration kinetics of PFOS by *P. wilsoni* and compared our results with those for other aquatic animals.

## Materials and Methods

Sandworms identified as *P. wilsoni* (Park and Kim 2007) were obtained from an aquaculture farm and were acclimated to the laboratory environment for 4 days without feeding.

Cylindrical containers (polypropylene, 182 mm o.d., 141 mm high) with 4-mm-diameter holes in the bottom were packed from bottom to top with an approximately 25-mm-high layer of gravel (6–8 mm), 0.5-mm-mesh polyethylene netting, and an approximately 70-mm-high layer of gravel (3–6 mm). The gravel was too large to be ingested by the sandworms. It was essentially held in the containers, but the water entered and drained smoothly through the holes. The containers were placed in 200-L polyethylene tanks (630 mm × 850 mm × 382 mm high) to control the water temperature and water level. All the materials in contact with experimental water were rinsed with water and with aqueous methanol or methanol prior to use. As experimental water, we used filtered (nominal pore size, 0.1 µm) natural seawater (Sakurai et al. 2013). The lighting cycle was approximately 10-h light and 14-h dark.

We conducted exposure and control (non-exposure) treatments. A 7-day exposure period was followed by a 9-day depuration period. On day 0, about eight sandworms were introduced to each of 26 exposure containers and eight control containers (these included two extra containers for each treatment). The water level in the tanks was varied daily to mimic the tides as follows: the level was kept at ~10 cm above the gravel surface for 18 h overnight (high level), at the bottom of the container for 1 h in the morning (low level), and then at the gravel surface for 5 h.

The exposure medium was the interstitial seawater in the containers. For the exposure treatment, a standard methanol solution of potassium PFOS (100 mg/L, PFOS-002S, AccuStandard, New Haven, CT, USA) was diluted in two steps to make a stock solution of spiked seawater at a nominal PFOS concentration of 32 ng/L (as anion). This stock seawater was prepared daily and supplied to the tanks during the exposure period. For the control treatment, methanol-spiked seawater (0.35 ppm v/v, the same concentration as that used in the exposure treatment) was similarly prepared and supplied during the same period. The exposure concentration was set to allow a sufficient difference in the PFOS concentrations in sandworms between the exposure and control treatments, as well as to be as close as possible to environmentally relevant levels (Sakurai et al. 2010). On day 7, at the end of the exposure period, the sandworms in each exposure container were transferred to unspiked seawater in a new container in a new tank. The sandworms were not fed during the exposure period but were fed daily (0.02 g/container) with commercial fish food (Ambrose 800, Nippon Formula Feed Mfg. Co., Ltd., Japan) during the depuration period.

Normally, the temperature and pH of the interstitial water were monitored daily, and salinity was measured with a refractometer and the ammonia concentration was determined by the salicylate method every other day. The dissolved oxygen (DO) level in the interstitial water was measured in multiple exposure containers with a fluorescent-type sensor on days 9 and 11 during the periods of high- and gravel-surface-water-levels for a total of ~20 min.

Three sets of eight sandworms were sampled on day 0 from the pool of acclimated sandworms. Subsequently, three containers were randomly sampled on days 1, 3, 5, 7, 9, 11, 13, and 16 from the exposure treatment and on days 7 and 16 from the control treatment. All sandworms collected from each sampled container were combined and treated as a composite sample. Dead sandworms were removed from the sample. For each treatment, nine individuals were randomly selected from the samples, weighed, and then returned to the corresponding sample for subsequent analysis. The sandworm samples were then kept at −20 °C until analysis. On each day of sandworm sampling in each treatment and on day 1 in the control treatment, approximately 20-mL portions of interstitial water were collected from each of three or more randomly selected containers in the treatment and then combined. Water samples were stored at 6°C until analysis.

PFOS concentrations in the samples were determined by previously reported methods with modifications (Sakurai et al. 2013). Briefly, homogenized sandworm and ground fish-food samples were spiked with 5 ng of ^13^C_4_-PFOS, mixed with silica gel, and then extracted with 20% aqueous methanol with an accelerated solvent extractor. The extract was cleaned up with solid-phase-extraction cartridges and then concentrated. For water samples, a 10- to 100-mL aliquot of each sample was filtered through glass-fiber filters. The filtrate (dissolved phase) was spiked with 1 ng of ^13^C_4_-PFOS, extracted with a solid-phase-extraction cartridge, and concentrated. The particulate phase (residue on filter) was also analyzed for the day-7 and day-16 samples. Identification and quantitation were carried out by injecting an aliquot of the final concentrated extract into a liquid chromatograph connected to a triple-quadrupole type tandem mass spectrometer. The detection limits were 0.1 ng/g-wet and 0.09 ng/g for 1 g-wet of sandworm and 0.5 g of fish-food samples, respectively, determined based on replicate analyses. The detection limits for water samples, determined from the signal-to-noise ratio of 8 on chromatograms and from 3 times the standard deviation of the method blank values, ranged from 0.6 to 2 ng/L for the samples (30–100 mL) designated as below the detection limit. A method blank was determined for each analysis batch, and the average blank value was subtracted from the sample values.

We analyzed the uptake and depuration of PFOS by the sandworms on the basis of a first-order-kinetics equation involving the whole-body PFOS concentration (Eq. 1) (Arnot and Gobas 2004):

1 $${{{\text{d}}C_{{\text{b}}} \left( t \right)} /{{\text{d}}t}} = k^{\prime}_{{{\text{resp}}}} C_{{{\text{dis}}}} - \left( {k^{\prime}_{{\text{e}}} + k^{\prime}_{{\text{m}}} + k_{{\text{g}}} } \right)C_{{\text{b}}} \left( t \right)$$

where *C*_b_(*t*) is the PFOS concentration in the sandworm as a function of time *t*; $C_{{{\text{dis}}}}$ is the dissolved concentration of PFOS in seawater; $k_{{{\text{resp}}}}$ is the rate constant for respiratory uptake, $k_{{{\text{e}}}}$ is the elimination rate constant, and $k_{{{\text{m}}}}$ is the rate constant for depuration due to metabolism (prime symbols indicate apparent values); and *k*_g_ is the growth rate constant of the exponential growth model of the sandworm, for which respective values were used for the exposure and depuration periods (*k*_g[E]_ or *k*_g[D]_). The apparent rate constants ($k^{\prime}_{{{\text{resp}}}}$ and [$k^{\prime}_{{\text{e}}} \, + \,k^{\prime}_{{\text{m}}}$]) were estimated by fitting the data to Eq. 1 (Sakurai et al. 2013) and were then corrected for the 1-h daily low-water-level period, during which uptake, elimination, and metabolic depuration were assumed not to occur (Eq. 2):

2 $$k = \left( {{{24} / {23}}} \right)k^{\prime}\quad ({\text{for}}\;k_{{{\text{resp}}}} ,\,\,k_{{\text{e}}} \;{\text{and}}\;\,k_{{\text{m}}} )$$

Depuration half-life (*t*_1/2_) was calculated as (ln 2)/(*k*_e_ + *k*_m_). The absorption efficiency of PFOS at the respiratory surfaces was estimated according to Eq. 3, by assuming that the respiratory uptake of PFOS was proportional to that of oxygen (O_2_) (Sakurai et al. 2013):

3 $$\alpha ^*\; = \;k_{{{\text{resp}}}} DO/r$$

where α* is the respiratory absorption efficiency of PFOS relative to that of O_2_, and *r* is the body-mass-specific O_2_ consumption rate of the sandworm. The uncertainties of the estimated parameters were estimated based on the propagation of uncertainty (Joint Committee for Guides in Metrology 2008).

We determined *r* by measuring the decrease of DO in a cylindrical airtight glass vessel packed with the gravel to a height of 95 mm. Eight sandworms were introduced to the vessel and acclimated overnight under a gentle flow of seawater. Then the vessel was filled with fresh seawater, capped, left for 2 h, and turned upside down gently (to avoid injuring the sandworms) to achieve homogeneous DO in the vessel. Consumed O_2_ mass was calculated from the difference between the DO levels at the start and end of the measurement multiplied by the volume of water in the vessel. Three measurements were made using different sets of individuals (average mass, 0.42 ± 0.11 g-wet), and the control value determined using a vessel containing only gravel and seawater was subtracted.

## Results and Discussion

During the experiment, the interstitial water in the containers had an average ± SD (both treatments) temperature of 17.1 ± 0.2°C, salinity of 36 ‰ ± 0‰, pH of 8.1 ± 0.0, and NH_3_-N concentration of 0.01 ± 0.01 mg/L. The time-weighted average DO in the interstitial water was 91% of saturation, and this value was used for the kinetics calculations.

In both treatments, the average mass of the sandworms naturally decreased during the exposure period and increased during the depuration period. The sandworms weighed 0.44 ± 0.07 g-wet per individual at the start of the experiment. The *k*_g[E]_ values were estimated at −0.012 ± 0.018 and −0.023 ± 0.021, and the *k*_g[D]_ values were estimated at 0.020 ± 0.013 and 0.027 ± 0.015, for the exposure and control treatments, respectively (values after ± are standard error for *k*_g_). Mortality was 2.2% and 6.8% for the exposure and control treatment, respectively, during the experiment.

**Fig. 1:**
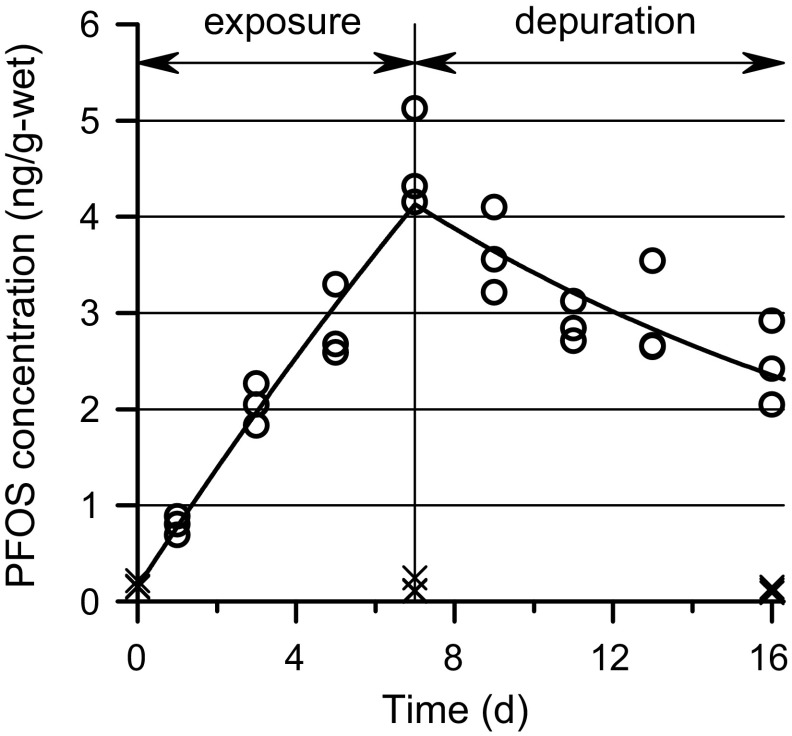
PFOS concentrations in sandworm samples from the exposure treatment (*circles*) and the control treatment and day-0 samples (*crosses*). The *curve* shows the first-order-kinetics model (Eq. 1) fitted to the exposure treatment data

In the exposure treatment, *C*_dis_ averaged 28 ± 21 ng/L during the exposure period. The PFOS concentration in the particulate phase (3.2 ng/L on day 7) made only a minor contribution to the PFOS concentration in the water, and the average *C*_dis_ was used for the kinetics analysis. The PFOS concentration was below the detection limit in the water samples from the control treatment and in the water samples from the depuration period of the exposure treatment. In the exposure treatment, *C*_b_ increased during the exposure period and decreased thereafter (Fig. 1). In the control treatment, *C*_b_ was stable throughout the experiment, and the average concentration (0.15 ng/g-wet) calculated from all the data, including the day-0 data, was subtracted from *C*_b_ in the exposure treatment before fitting to the model. The PFOS concentration was below the detection limit in the fish food.

We measured *r* to be 62 ± 9.8 µg-O_2_/(g-wet h), which is comparable to previously reported *r* values for this and related species. Empirical relationships, reported by Kristensen (1981), between *r* and the body mass of 3 *Nereis* sandworm species at a salinity of 20‰ and at 16°C predict a resting *r* of 42–70 µg-O_2_/(g-wet h) for a body mass of 0.42 g-wet. Yoshida (1972) reported the *r* of *P. nuntia vallata* as 33 µg-O_2_/(g-wet h) at a salinity of 32.6‰ and at 20 °C for individuals weighing 0.08–0.3 g. The lower value than the present study may be because Yoshida measured the rate in only seawater without gravel, which is not the normal habitat of this species.

We compared the values of the kinetics parameters obtained in this study (Table 1) to previously reported values for other aquatic species. The respiratory absorption efficiency of PFOS was estimated to be 0.11 that of O_2_. This value is low compared to values typically reported for hydrophobic neutral compounds in fish (Kobayashi et al. 2013; McKim et al. 1985), probably because of the presence of the charged sulfone group in PFOS. However, the value is higher than the values estimated for PFOS in several fish species (0.007–0.095) (Sakurai et al. 2013). The PFOS *k*_resp_ value was in the same range as reported whole-body values for several fish species and an oyster species [5.3–53 L/(kg-wet day)] (Jeon et al. 2010; Martin et al. 2003; Sakurai et al. 2013).

**Table 1:** Kinetics parameters for respiratory uptake and depuration of PFOS in the sandworm *Perinereis wilsoni* ^a^

Parameter	Unit	Point estimate	95% CI
*k*_resp_	mL g-wet^−1^ day^−1^	22	7.9–70
α*		11%	3.9%–30%
*k*_e_ + *k*_m_	day^−1^	0.047	0.027–0.068
$t_{{1/2}}^{{b}}$	day	15	10–26

^a^Average mass = 0.44 g-wet at the start of the experiment

^b^ $$t_{{1/2}} = \left( {\ln {\text{ }}2} \right)/\left( {k_{{\text{e}}} + k_{{\text{m}}} } \right)$$

The value of *k*_e_ + *k*_m_ for PFOS corresponded to a half-life of 15 days and was similar to the value for a freshwater oligochaete (0.038 day^−1^; Higgins et al. 2007) and the whole-body values for the several fish species (Martin et al. 2003; Sakurai et al. 2013) but was lower than that for the oyster (0.10–0.42 day^−1^; Jeon et al. 2010). The bioconcentration factor (BCF), calculated as *k*_resp_ divided by (*k*_e_ + *k*_m_), was 470, which is slightly lower than the range for the fish species (720–2800; Martin et al. 2003; Sakurai et al. 2013) and 1 order of magnitude higher than the value for the oyster (31–86; Jeon et al. 2010).
